# Correction: Yasunaga et al. Associations Among Developmental Coordination Disorder Traits, Neurodevelopmental Difficulties and University Personality Inventory Scores in Undergraduate Students at a Japanese National University: A Cross-Sectional Correlational Study. *Brain Sci.* 2025, *15*, 895

**DOI:** 10.3390/brainsci16020206

**Published:** 2026-02-10

**Authors:** Masanori Yasunaga, Ryutaro Higuchi, Keita Kusunoki, Naoto Mochizuki

**Affiliations:** 1 Health and Counseling Center, The University of Osaka, Toyonaka 560-0043, Japan; 2 Student Support Center, Bukkyo University, Kyoto 603-8301, Japan

**Error in Table**

In the original publication [1], there was a mistake in Table 1 as published. The value reported for “Concentration” was incorrectly shown as “0.000”. The corrected Table 1 appears below. The authors state that the scientific conclusions are unaffected. This correction was approved by the Academic Editor. The original publication has also been updated.

**Text Correction**

Additional minor revisions were also made to improve clarity and ensure consistency with the Academic Editor’s recommendations. Specifically, (i) *p*-value notation was standardized across the relevant tables (e.g., “*p* < 0.001” rather than “*p* = 0.000”); (ii) a note was added to Table 2 to explicitly state that Spearman’s rank correlation coefficients were reported; (iii) wording in Table 3 and Section 3.3.1 was revised from “predicting” to “examining associations” to avoid unintended predictive implications in this cross-sectional study; (iv) Table 4 title/wording was adjusted to avoid describing the results as “hierarchical” in the table heading, while keeping the model specification, reported statistics, and interpretation unchanged; (v) the supplementary table reference was corrected for consistency (Supplementary Table S2); (vi) in Section 3.3.2, brief explanatory sentences were added to interpret the VIF values (confirming the absence of problematic multicollinearity) and to clarify the interpretation of the regression pattern in which AAC-Q becomes non-significant after adding ASD- and ADHD-related difficulties (suggesting possible overlap); and (vii) minor wording adjustments were made in the Discussion (Section 4.2) and Conclusions (Section 5) to improve clarity and maintain consistency with the cross-sectional design. This correction was approved by the Academic Editor. The original publication has also been updated.

The authors state that the scientific conclusions are unaffected. This correction was approved by the Academic Editor. The original publication has also been updated.

## Figures and Tables

**Table 1:** Comparison of Assessment Results between Groups with and without DCD Traits.

Outcome Measure	Group with DCD Traits	Group Without DCD Traits (n = 488)		
	(n = 39)			
	Median	Median	*p*-Value	Effect Size
	(25th–75th Percentile)	(25th–75th Percentile)		
Sex, n (%) ^†^			0.055 ^†^	0.091
Male	16 (41.0)	284 (58.2)		
Female	23 (59.0)	204 (41.8)		
Age	20 (19–22)	21 (18–23)	0.808 ^‡^	0.11
AAC-Q				
Total score	35 (33.0–37.0)	19(15.0–23.0)	<0.001 ^‡^	0.45
ADHD				
Total score	13 (10.0–20.0)	4 (2.0–7.0)	<0.001 ^‡^	0.36
Concentration	1 (0–1.0)	1 (0–1.0)	0.555 ^‡^	0.03
Inattention	3 (1.0–5.0)	0 (0–2.0)	<0.001 ^‡^	0.29
Impulsivity	3 (1.0–4.0)	0 (0–1.0)	<0.001 ^‡^	0.33
Sleep	1 (0–2.0)	0 (0–1.0)	<0.001 ^‡^	0.18
Planning	2 (0–3.0)	0 (0–1.0)	<0.001 ^‡^	0.24
Clumsiness	3 (2.0–5.0)	1(0–2.0)	<0.001 ^‡^	0.33
Organization	2 (1.0–3.0)	1(0–1.0)	<0.001 ^‡^	0.25
ASD				
Total score	17 (9.0–23.0)	6 (2.0–11.0)	<0.001 ^‡^	0.27
Autism	10 (4.0–16.0)	3 (1.0–6.0)	<0.001 ^‡^	0.27
Interpersonal	7 (3.0–9.0)	2 (1.0–5.0)	<0.001 ^‡^	0.2
SLD				
LDSP7 total score	11 (9.0–14.0)	9 (7.0–11.0)	<0.001 ^‡^	0.22
SCLD10 total score	14 (11.0–18.0)	11 (10.0–14.0)	<0.001 ^‡^	0.14
UPI				
Total Score	14 (5.0–21.0)	5 (1.0–10.0)	<0.001 ^‡^	0.23
suicidal ideation	0.00	0.00	<0.001 ^‡^	0.23
Mental Health				
stress	3 (2.0–3.0)	2 (2.0–2.0)	<0.001 ^‡^	0.18

† chi-square test, effect size = Cramér’s V; ‡ Mann–Whitney U test, effect size = r. AAC-Q: Adolescents and Adults Coordination Questionnaire; ADHD: Attention Deficit Hyperactivity Disorder Difficulty Scale (Short Version); ASD: Autism Spectrum Disorder Difficulty Scale (Short Version); LDSP7: Learning Difficulty Scale for Postsecondary Students (7-item); SCLD10: Scale for Childhood Learning Difficulties (10-item); UPI: University Personality Inventory; MT: Mental health.
